# Correlation of Burnout Syndrome with Emotional Intelligence among Clinicians at Workplace

**DOI:** 10.12669/pjms.41.1.9725

**Published:** 2025-01

**Authors:** Aliena Badshah, Adnan Yousaf, Usman Mehboob, Muhammad Babar Khan

**Affiliations:** 1 Aliena Badshah, Department of Medicine, Khyber Teaching Hospital, Peshawar, Pakistan; 2 Adnan Yousaf, Dr. Maqbali Khan Hospital, Peshawar, Pakistan; 3 Usman Mehboob, Khyber Medical University, Peshawar, Pakistan; 4 Muhammad Babar Khan, Khyber Medical College, Peshawar, Pakistan

**Keywords:** Burnout syndrome, Emotional intelligence, Maslach Burnout Inventory, Emotional Exhaustion, Personal Accomplishment, Depersonalization, Schutte Emotional Intelligence Scale

## Abstract

**Objective:**

To determine the correlation of burnout syndrome with emotional intelligence at workplace.

**Methods:**

This cross-sectional study was conducted in six public sector hospitals of Khyber Pakhtunkhwa between June and November, 2022. Male and female clinicians from Medicine, Surgery, Obstetrics / Gynaecology, Paediatrics Medicine, Paediatric Surgery, Gastroenterology, Anesthesiology, Psychiatry, Radiology, Dermatology, Ophthalmology, Otorhinolaryngology, Nephrology, Urology, Neurology, Neurosurgery, Orthopaedics, Endocrinology, Rheumatology and Cardiology were included in the study. The study was initiated after ethical approval from ethical review board of Khyber Medical University. Informed consent was taken from all study participants. They were given a proforma to fill. The proforma was divided into three categories; clinician demographics, Maslach Burnout inventory, and Schutte Emotional Intelligence Scale. All questions on the Maslach Burnout inventory and Schutte Emotional Intelligence Scale pertained to their duty hours from 8am to 4pm. The Maslach Burnout Inventory was stratified into three sub-scales of emotional exhaustion, personal accomplishment and depersonalization. Results were compiled in SPSS 25. Correlation between the three strata of MBI was made with SEIS and stratified on the basis of gender, specialty, designation, institute, and clinical experience.

**Results:**

Emotional exhaustion has a negative correlation at Spearman correlation = -0.272 with SEIS; Personal accomplishment has a positive correlation at Spearman correlation = 0.402; Depersonalization has a negative correlation with SEIS at Spearman correlation = -0.349.

**Conclusions:**

There is a weak correlation between burnout syndrome and emotional intelligence among clinicians. Further studies need to be conducted on a larger scale to explore the reasons behind this weak correlation.

## INTRODUCTION

Health care workers are continuously exposed to stress related to work, duty hours, patient care, frequently changing administrative policies, teaching to under- and post-graduate students and inter-colleague relations. Due to the ever-increasing demands of modern-day health care practices imposed by the society, the clinicians are feeling exhausted and this exhaustion is evident in their personalities.^1^ The burden of burn out can propagate into their personal lives too.

Recently, the increasing burnout among clinicians has raised concerns over its impact on health care delivery which can ultimately put patient safety at stake. Burn out among clinicians can lead to job dissatisfaction and ultimately a desire to quit their jobs.^2^ Depersonalization among burnt out clinicians can also cause poor doctor patient interaction.^3^

Burn out can also influence the emotional intelligence of the clinician. An individual’s personal attributes play an important role in dealing with difficult situations. Emotional intelligence, which is one of those attributes, also comes into play in association with physician burnout. It is a personality trait.^3^,^4^ An emotionally intelligent health care worker might feel less burnt-out as compared to a less emotionally intelligent personal.

Any clinician’s interaction with other colleagues and patients can strongly be influenced if they are feeling burnt out.^5^ Literature is prosperous on the individual topics of burnout syndrome and emotional intelligence; however sparse on the association between these two entities. It would be difficult to develop a cause-effect relation between burnout syndrome and emotional intelligence.

Some studies have taken emotional intelligence as autonomous variable and burnout as the reliant variable with demonstration of 8.2% variability in burnout syndrome.^6^ However even if an association is established between the two, targeted steps need to be taken to groom emotional intelligence in health care workers that are prone to develop burnout syndrome.

The aim of current thesis was to demonstrate the correlation between burnout syndrome and emotional intelligence at workplace. Previous research has been carried out on association of emotional intelligence and burnout among nurses, but an elaboration of the correlation between these two entities among clinicians has not been undertaken yet.^6^,^7^ A study on the correlation of these two entities among clinicians will enable us to better address this issue in the form of workshops on Emotional Intelligence and burnout especially for disciplines more at stake of burnout due to low emotional intelligence and / or addressal at the level of administration resulting in job relaxation to avoid burnout. The objective was to determine the Correlation of burnout syndrome with emotional intelligence among clinicians at workplace.

## METHODS

This cross-sectional study was conducted between June and November, 2022. Data was collected from six public sector tertiary care hospitals of Khyber Pakhtunkhwa. These include: Khyber Teaching Hospital (KTH), an 1800 bedded hospital located in Peshawar; Hayatabad Medical Complex (HMC), a 1300 bedded hospital located in Peshawar; Lady Reading Hospital (LRH), a 1500 bedded hospital located in Peshawar; Institute of Kidney Diseases (IKD), a 200 bedded hospital specific for kidney diseases situated in Peshawar, Mardan Medical Complex (MMC), a 520 bedded hospital situated in Mardan; and Ayub Teaching Hospital (ATH), a 1300 bedded hospital located in Abbottabad.

The sample size as calculated by WHO sample size calculator keeping confidence interval at 95%, p-value < 0.05, absolute precision 0.01 and relative precision 0.2 was 357 + 10% for any losses = 400. Random sampling technique was used.

Clinicians from Medicine, Surgery, Obstetrics / Gynaecology, Paediatrics Medicine, Paediatric Surgery, Gastroenterology, Anesthesiology, Psychiatry, Radiology, Dermatology, Ophthalmology, Otorhinolaryngology, Nephrology, Urology, Neurology, Neurosurgery, Orthopaedics, Endocrinology, Rheumatology and Cardiology were included in the study. Paediatric surgery, otorhinolaryngology, neurosurgery, and rheumatology each had fewer clinicians than these other specialties; hence, statistically significant data could not have been obtained for these fields. Paediatric surgery, otorhinolaryngology, and neurosurgery were added to the surgical specialty, while rheumatology was added to the medical specialty.

### Inclusion & Exclusion Criteria:

Male and female clinicians (Assistant Professors, Associate Professors, Professors) from public sector tertiary care hospitals of Khyber Pakhtunkhwa during their designated hospital duty hours of 8am to 4pm were included in the study. Trainee medical officers (TMOs) and house officers (HOs) were excluded from the study. Clinicians from private hospitals were also excluded from the study.

### Ethical Approval:

After ethical approval from Institutional Research and Ethics Board (IREB) of Khyber Medical University via Ref. No: 1-10/IHPER/MHPE/KMU/22-45, dated September 19, 2022 data collection was initiated.

The questionnaire / proforma was distributed among clinicians mentioned above through WhatsApp using google forms. The questionnaire comprised of clinicians’ demographic and work place related data, Maslach Burnout Inventory (MBI) and Schutte Emotional Intelligence Scale (SEIS). The details obtained from google forms were then extrapolated to excel sheet wherefrom data was analyzed. MBI is an open access questionnaire available online. Consent from Nicola Schutte who has devised the SEIS was taken.

The participants were stratified into their respective disciplines. Data from participants from each discipline was analyzed separately. Burnout was classified into high, moderate and low level of burnout based on scores achieved at emotional exhaustion, depersonalization, and personal accomplishment domains of burnout syndrome.

Emotional intelligence was scored on the Schutte Emotional Intelligence Scale. The scores of each clinician on MBI were compared to scores obtained on SEIS and correlation between the respective scores from both scales was made. This was done using Spearman correlation.

The Cronbach’s alpha coefficient of estimated internal consistency for emotional exhaustion sub-scale component of the MBI was 0.949; that for personal accomplishment sub-scale was 0.927; that for depersonalization was 0.787, and that for SEIS was 0.716. Hence the data yielded from the questionnaire is deemed reliable.

## RESULTS

The study has yielded some interesting results. The demographic details of clinicians included in the study are highlighted in Table-I. Stratification on the basis of gender (male / female), designation (assistant professor, associate professor, professor), institute (KTH, HMC, LRH, IKD, MMC, ATH), age group (31-40, 41-50, 51-60 years), total duration in institute (0-5, 6-10, 11-15, 16-20, 21-25 years), and duration of stay in same designation (<5 years, >5 years) is done in the table below. Table-II highlights the specialty-wise distribution of clinicians while Table-III depicts the correlation of MBI sub-scales with SEIS.

**Table-I:** Demographic analysis of study sample.

Demographics			

		Frequency (n)	Percent (%)
Gender	Male	283	70.75
	Female	117	29.25
Designation	Assistant Professor	241	60.25
	Associate Professor	105	26.25
	Professor	54	13.50
Institute	KTH	115	28.75
	HMC	93	23.25
	LRH	18	4.50
	IKD	24	6.00
	MMC	58	14.50
	ATH	92	23.00
Age group	31-40 years	127	31.75
	41-50 years	180	45.00
	51-60 years	93	23.25
Duration in Institute	0-5 years	154	38.5
	6-10 years	102	25.5
	11-15 years	77	19.25
	16-20 years	41	10.25
	21-25 years	26	6.5
Duration of stay in same designation	Less than or equal to 5 years	382	95.50
	More than 5 years	18	4.50

**Table-II:** Specialty-wise distribution of clinicians.

		Frequency (n)	Percentage (%)
Specialty	Medicine	60	15.00
	Surgery	49	12.25
	Paediatrics	25	6.25
	Obstetrics & Gynecology	54	13.50
	Radiology	27	6.75
	Ophthalmology	12	3.00
	Oncology	3	0.75
	Cardiology`	23	5.75
	Dermatology	9	2.25
	Neurology	1	0.25
	Orthopedics	13	3.25
	Endocrinology	4	1.00
	Neurosurgery	17	4.25
	Pathology	17	4.25
	Nephrology	19	4.75
	Urology	8	2.00
	Gastroenterology	19	4.75
	Anesthesiology	26	6.50
	Pulmonology`	6	1.50
	Psychiatry	8	2.00
	Total	400	100.00

**Table-III:** Overall correlation of MBI sub-scales with SEIS (n = 400).

S. No	MBI sub-scale	Spearman Correlation with SEIS
1.	Emotional Exhaustion	-0.272**
2.	Personal accomplishment	0.402**
3.	Depersonalization	-0.349**

** . Correlation is significant at 0.01 level (2- tailed).

The gender-wise correlation of MBI sub-scales with SEIS among clinicians are shown in Table-III while Table-V depicts the correlation of MBI sub-scales with SEIS among different cadres of clinicians. Table-VI highlights the correlation between burnout syndrome sub-scales and EI in six public sector hospitals of KPK. Correlation of burnout sub-scales and EI among different clinicians is depicted in Table-VII.

**Table-IV:** Gender-wise correlation of MBI sub-scales with SEIS among clinicians

S. No.	MBI sub-scale	Spearman correlation with SEIS among male clinicians	Spearman Correlation with SEIS among female clinicians
1.	Emotional exhaustion	-0.303**	-0.178
2.	Personal accomplishment	0.404**	0.391**
3.	Depersonalization	-0.433**	-0.141

** Correlation is significant at the 0.01 level (2-tailed).

**Table-V:** Correlation of MBI sub-scales with SEIS among different cadres of clinicians.

S. No.	MBI sub-scale	Spearman Correlation with SEIS among Assistant Professors	Spearman Correlation with SEIS among Associate Professors	Spearman Correlation with SEIS among Professors
1.	Emotional exhaustion	-0.328**	-0.150	-0.185
2.	Personal accomplishment	0.496**	0.272**	0.169
3.	Depersonalization	-0.443**	-0.055	-0.297*

** . Correlation is significant at the 0.01 level (2-tailed).

* . Correlation is significant at the 0.05 level (2-tailed).

**Table-VI:** Correlation of MBI sub-scales with SEIS across the six public sector hospitals of KPK

S.No	MBI sub-scale	Spearman correlation with SEIS in KTH	Spearman correlation with SEIS in HMC	Spearman correlation with SEIS in LRH	Spearman correlation with SEIS in IKD	Spearman correlation with SEIS in MMC	Spearman correlation with SEIS in ATH
1.	Emotional exhaustion	-0.324**	-0.137	-0.190	-0.553**	-0.336*	-0.248**
2.	Personal accomplishment	0.517**	0.494**	0.618	0.734**	0.206	0.233*
3.	Depersonalization	-0.297**	-0.537**	-0.377	-0.658**	-0.085	-0.262

** . Correlation is significant at the 0.01 level (2-tailed).

* . Correlation is significant at the 0.05 level (2-tailed).

**Table-VII:** Correlation of MBI sub-scales with SEIS among different clinicians

S. No.	Clinicians	Spearman correlation of MBI sub-scales with SEIS		

		Emotional Exhaustion	Personal accomplishment	Depersonalization
1.	Physicians	-0.140	0.255*	-0.155
2.	Surgeons	-0.589**	0.568**	-0.555**
3.	Paediatricians	-0.471*	0.452*	-0.311
4.	Obstetricians & Gynecologists	-0.107	0.268*	-0.129
5.	Radiologists	0.096	0.311	0.429*
6.	Cardiologists	0.292	0.070	-0.235
7.	Nephrologists	-0.065	0.529*	-0.505*
8.	Anesthesiologists	-0.251	0.213	-0.183

** . Correlation is significant at the 0.01 level (2-tailed).

* . Correlation is significant at the 0.05 level (2-tailed).

**Fig.1:**
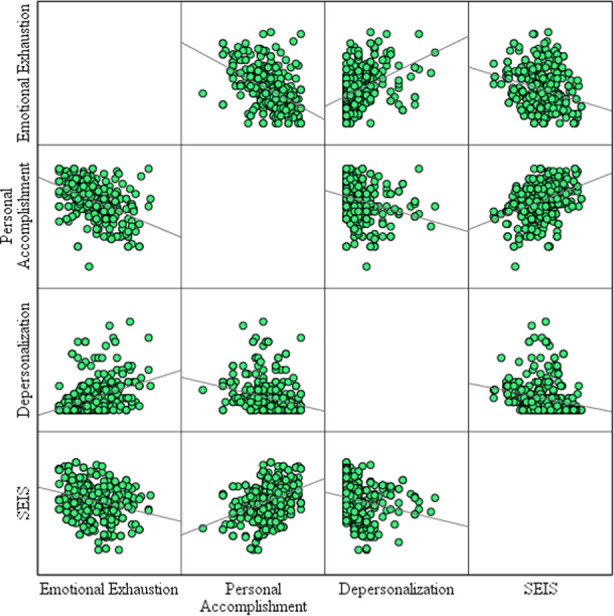
Diagrammatic representation of Spearman Correlation between MBI sub-scales and EI.

The correlation of MBI sub-scales and SEIS among clinicians based on their clinical experience in same designation is elaborated in Table-VIII while Fig.1 depicts the diagrammatic representation of Spearman correlation between MBI sub-scales and EI.

**Table-VIII:** Correlation of MBI sub-scales with SEIS based on clinical experience of clinicians

S. No.	MBI sub-scale	Clinicians with ≤ 5 years experience	Clinicians with > 5 years experience
1.	Emotional exhaustion	-0.272**	-0.333
2.	Personal accomplishment	0.391**	0.720**
3.	Depersonalization	-0.358**	-0.054

** . Correlation is significant at the 0.01 level (2-tailed).

## DISCUSSION

In order to examine the harm or repercussions of burnout, the majority of studies among health care professionals on feeling burnt out at work-place have employed a mode based on diseases.^8^ The fact that there are 14 times as many articles on negative states as there are on positive states serves as an example of the general negative bias.^9^

To reduce the occurrence and impact of EI among health care workers especially doctors, the term emotional intelligence should be familiarized among patients and doctors and openly in literature so that people can appreciate the havoc it can play with any individual’s intact state of mind.^10^ Ways and means need to be devised to measure EI and also develop an operational definition for it. There are four components to the concept of EI: appraisal of one’s own emotions, appraisal of others’ emotions, regulation of one’s emotions, and utility of one’s emotions. Additionally, the concept aims to portray the variety of approaches people use to controlling their emotions. Emotional intelligence is therefore a prerequisite for key skills including self-awareness, self-control, empathy, creativity, and self-confidence. 11

According to studies, emotional intelligence is linked to one’s sense of stress, problem-solving skills, and subjective well-being.^12^,^13^ It has been demonstrated that emotional intelligence correlates with patient approval, the frequency of medical miscalculation, communication abilities, and the performance of medical staff. As research continues on EI, it has been incorporated into the fields of medical education as well as clinical psychology in the form of empirical studies.^14^ EI is an indispensable pillar required for proper functioning of the society. It is interesting that so few researches have looked at the connection between emotional intelligence and professional burnout among healthcare professionals in medical practice, despite the fact that emotional intelligence has a noteworthy and profoundly hopeful placement.

The results of this study have yielded some interesting information on the correlation of burnout syndrome with emotional intelligence. The overall scrutiny of burnout syndrome on the MBI and that of emotional intelligence on SEIS has demonstrated that there is a correlation between the two. It has also elaborated that emotional exhaustion and depersonalization sub-scales are in inverse relation to emotional intelligence, whereas personal accomplishment is in direct relationship with emotional intelligence. Although the study has succeeded in discovering the inverse and direct correlations of different aspects of burnout syndrome with emotional intelligence, the correlation; however, is a weak one. One likely possibility could be the inability of clinicians to respond to the questionnaire while keeping other social stressors apart from work place aside.

The effects of emotional intelligence on burnout syndrome, job satisfaction, consideration, and communication skills of trainees in the field of intensive care unit were examined by Moraes AGD et al.^15^ 26 trainees participated in their study, with an average Emotional Intelligence score of 158. 23 of these study participants had Burnout Syndrome. The findings demonstrated a positive association between high emotional intelligence and low levels of emotional inertia (correlation coefficient -0.685, p 0.001) and depersonalization (correlation coefficient -0.506, p = 0.008). Additionally, high levels of EQ were associated with greater levels of empathy (correlation coefficient 0.605, p 0.001), communication skills (correlation coefficient 0.591, p = 0.001), and professional satisfaction (correlation coefficient 0.632, p 0.001). The research concluded that intensive care trainees are burnt out at a high rate. Conclusions made from this study hi-lighted that EI is related to satisfaction at work place, empathy and better communication skills.

The effects of workplace based emotions on nursing students during their clinical rotations in hospitals were seen in a meta-correlation analysis.^16^ One hundred and seventy one nursing students who were enrolled in a 4-year program with practical training that started almost six months prior to the commencement of the inquiry were the subject of the survey. On a scale of 1 to 5, the professional feelings category received an average rating of 3.17. Professional sentiments had a substantial impact on professional weariness for nursing students (F = 15.763, p 0.001). The relationship between working emotions and burnout syndrome has been significantly mediated by the level of emotional intelligence (F = 15.345, p 0.001).

Fifty-six doctors from different disciplines, including surgeons, were evaluated by Swami et al. in 2013 for their emotional intelligence and burnout syndrome.^17^ The results revealed a significant association between perceived stress and burnout syndrome and a negative correlation between emotional intelligence. Additionally, there was a poor link between perceived stress and emotional intelligence. According to the examination of the mediation, emotional intelligence’s impact on burnout syndrome was moderated by the capacity to recognize stress.^18^ Results of this study conform to the findings of our study in which surgeons demonstrated a clear correlation between the three sub-scales of MBI with SEIS. In our study, the most obvious correlation in accordance with our hypothesis was found among surgeons. Further studies need to be conducted on the differences between emotional responses of surgeons and other clinicians.

Aim of our research was to understand how clinicians from various specialties’ onset and progression of burnout syndrome may have any correlation with emotional intelligence. More particularly, it explores whether among professionals with expertise in various clinical domains, the amount of emotional intelligence and its components—such as wellbeing, willpower, perceptivity, and amiability— have any correlation with burnout syndrome and whether these variables can lower its level. It investigates if the demographic background of professionals has an impact on their degrees of emotional intelligence and burnout syndrome. This includes their gender, designation, institute, and clinical experiences.

Our study demonstrated that clinicians with less experience e.g. assistant professors had more burnt out than those with greater experience. This means, clinicians who were in age group <45 years felt more burnt out than those who were in >45 years age group. This could be attributed to greater work load on the junior clinicians. Other studies have also demonstrated similar results. The factor “Personal achievements” performed poorly for those with 10 to 19 years of experience, increasing the risk of burnout. The people who “belong” to that age group were therefore disillusioned by their achievements in both the personal and professional spheres.^19^,^20^ At the same time, those who reported being “completely unhappy” with their economic salaries tended to have reduced performance at the factor “Personal achievements” and, as a result, greater levels of Burnout Syndrome. The fact that the same individuals scored high on the category “Emotional Exhaustion” is worth mentioning because it favors this conclusion.^21^

The process of emotionality involves identification of stress, most bothersome aspect of that stress and then addressal of stress in the form of change in behavioral outlook, self control and self esteem. It is important to note the Trait Emotional Intelligence Que-Short Form’s predictive value for the Maslach Burnout Inventory at this point because it was discovered that if the Trait Emotional Intelligence Que-Short Form score increased by one unit, the Maslach Burnout Inventory score decreased by 17 units.^22^,^23^ Prior to this study, there were no comparable studies conducted in Greece. Because of this, the current findings provide new information that calls for more research in order to fully understand this occurrence. The previous hypothesis was supported by inverse correlation between burnout syndrome and EI among professionals working in rehabilitation units, the ability of EI to diagnose burnout syndrome, and the impact of demographics of study sample on this relationship of burnout with EI.^24^

A study conducted among medical teachers in Lahore, Pakistan also concluded that emotional intelligence and burnout have a negative correlation. However, it encompassed teachers of pre-clinical years who are involved in teaching to under-graduate medical students only and are not exposed to post-graduate teaching and / or hospital related work load.^25^

The present study’s findings are consistent with the basic hypothesis that emotional intelligence is adversely correlated with burnout syndrome, which can be anticipated and mitigated.

### Limitations:

The study examines the interaction between burnout and emotional intelligence at the ‘workplace’ during ‘designated duty hours’ of 8 a.m. to 4 p.m., Monday through Friday; however, the clinician may be bearing the brunt of other social stressors outside the workplace and these duty hours, hence their influence may have influenced the results of this work-based study. Also, any existing mental or psychiatric illness or disorder, as well as any chronic illness, can negatively impact both emotional intelligence and the clinician’s tendency to develop burnout syndrome. This could not be practically assessed before data collection.

The study was carried out in Khyber Pakhtunkhwa’s public tertiary care facilities. The relationship between burnout and emotional intelligence in private sector hospitals is seldom highlighted. Another study that includes private sector hospitals could produce intriguing results, and a comparison of the outcomes from public versus private hospitals will produce findings that are statistically significant. The situation in Pakistan will also be more clearly defined by studies conducted in other provinces.Although the study found a link between burnout syndrome and emotional intelligence, it was unable to prove a cause-and-effect relationship.

## CONCLUSION

The current study has concluded that there is a negative correlation between emotional exhaustion and depersonalization sub-scales of burnout syndrome with emotional intelligence, and a positive correlation between personal accomplishment sub-scale of burnout syndrome and emotional intelligence. However, it has not been able to elicit a strong correlation. Whether negative or positive, the correlation is weak. Reasons behind this weak correlation need to be probed.

The study concludes that there is no difference in the responses gathered from male and female clinicians, hence there is no significant variation in the correlation between burnout syndrome and emotional intelligence in either gender.

The correlation between burnout syndrome and emotional intelligence is more evident in the assistant professors. This could be attributed to their greater work load and lack of experience in stress management. Further interventional studies might shed further light on this issue. The younger clinicians need to be enabled to manage work place related stresses more firmly.

### Future Research:

Research needs to be carried on cause-and-effect relation of burnout syndrome and emotional intelligence, correlation of the two entities in public sector versus private sector hospitals, and such studies should be conducted across Pakistan to see variability in the results.

### Recommendations:

Recognition of physicians who are at risk for burnout and/or have low emotional intelligence, job relaxation, flexible duty hours, and referral to psychotherapy for such professionals may be helpful. The inclusion of workshops on enhancing emotional skills of trainee medical officers during post-graduate training might play an important and positive role in inculcating basic skills related to workplace stress management and handling.

### Authors’ contribution:

**AB:** Concept, Data collection, Compilation of results, formatting of the article, responsible for accuracy and integrity of the work. **AY:** Data collection, Data analysis, Critical appraisal, and Discussion Writing. **UM:** Overall compilation of the article, Critical Review. **MBK:** Data compilation, Bibliography All authors have approved the final version and are accountable for integrity of the study.
